# Detection of Crosslinks within and between Proteins by LC-MALDI-TOFTOF and the Software FINDX to Reduce the MSMS-Data to Acquire for Validation

**DOI:** 10.1371/journal.pone.0038927

**Published:** 2012-06-18

**Authors:** Christopher A. G. Söderberg, Wietske Lambert, Sven Kjellström, Alena Wiegandt, Ragna Peterson Wulff, Cecilia Månsson, Gudrun Rutsdottir, Cecilia Emanuelsson

**Affiliations:** Department of Biochemistry and Structural Biology, Center for Molecular Protein Science, Institute for Chemistry and Chemical Engineering, Lund University, Lund, Sweden; University of Arkansas for Medical Sciences, United States of America

## Abstract

Lysine-specific chemical crosslinking in combination with mass spectrometry is emerging as a tool for the structural characterization of protein complexes and protein-protein interactions. After tryptic digestion of crosslinked proteins there are thousands of peptides amenable to MSMS, of which only very few are crosslinked peptides of interest. Here we describe how the advantage offered by off-line LC-MALDI-TOF/TOF mass spectrometry is exploited in a two-step workflow to focus the MSMS-acquisition on crosslinks mainly. In a first step, MS-data are acquired and all the peak list files from the LC-separated fractions are merged by the FINDX software and screened for presence of crosslinks which are recognized as isotope-labeled doublet peaks. Information on the isotope doublet peak mass and intensity can be used as search constraints to reduce the number of false positives that match randomly to the observed peak masses. Based on the MS-data a precursor ion inclusion list is generated and used in a second step, where a restricted number of MSMS-spectra are acquired for crosslink validation. The decoupling of MS and MSMS and the peptide sorting with FINDX based on MS-data has the advantage that MSMS can be restricted to and focused on crosslinks of Type 2, which are of highest biological interest but often lowest in abundance. The LC-MALDI TOF/TOF workflow here described is applicable to protein multisubunit complexes and using ^14^N/^15^N mixed isotope strategy for the detection of inter-protein crosslinks within protein oligomers.

## Introduction

Chemical crosslinking of proteins combined with mass spectrometric analysis of the crosslinked products can be used to obtain low-resolution structural information for proteins, and novel information on protein-protein interactions [1], [2], [3]. Of special interest is the possibility to gain insight into the organization of protein complexes, both stable and transient complexes, for which the structure of the individual subunits may or may not be structurally determined [4]. However, in a tryptic digest of a crosslinked protein sample, informative inter-peptide crosslinks (type 2 crosslinks) are in low abundance compared to unmodified peptides and dead-end and intra-peptide crosslinks (type 1 and type 0, nomenclature according to [5]). Crosslink detection and the unambiguous confident assignment of peaks to crosslinks remain a challenge when investigating more than one protein, especially large proteins with long amino acid sequences that generate many different tryptic peptides. The challenge becomes even greater when it comes to transient protein complexes compared to stable protein complexes. The amount of complexes present at any time-point is very low in weak and transient protein-protein interactions [6], [7], and the percentage of informative crosslinks decreases even further.

To address the problem of recognizing crosslinks in very complex peptide mixtures, isotope-labeled crosslinking reagents are useful [8], and two lysine-specific crosslinkers, 3,3’-dithiobis(sulfosuccinimidylpropionate) (DTSSP) and bis(sulfosuccinimidylsuberate) (BS^3^), are commonly used in crosslinking studies. Both contain two sulfosuccinimide esters that react with the primary amines in the side chain of lysines and in the protein N-terminus. Additionally, DTSSP but not BS^3^ contains a cleavable disulfide bond. The commercially available isotope-labeled reagents are mixtures of 50% light and 50% heavy reagent with the heavy reagent being ∼8 Da (DTSSP) and ∼12 (BS^3^) Da heavier than the light reagent. Light and heavy crosslinking molecules react with and crosslink two primary amines within close spatial proximity. After digestion of the crosslinked protein(s), the crosslink between these two amines will appear in the MS spectrum as isotope doublet peaks (mass differences Δ8 and Δ12, for DTSSP and BS^3^), as a signature with which peptides that are modified by the isotope-labeled crosslinker can be recognized.

Despite isotope-labeling and other advancements in the design of crosslinking reagents [9] the major bottleneck in the identification of crosslinks has been the data analysis of crosslinking mass spectrometry data. This field is developing intensively now (for a recent review of software developed to assist data analysis see [10]), with new types of software continuously appearing, such as recently CrossWork [11] and StavroX [12]. Many programs are based on the acquisition of a very large number of MSMS-data to unambiguously identify the crosslinks.

Here we show how the advantages offered by off-line LC-MALDI-TOF/TOF mass spectrometry can be used in a quick MS-screening for crosslinks in a two-step workflow, which serves to limit the acquisition and analysis of MSMS-data necessary for crosslink identification. Using LC-MALDI-MS with the software FINDX it is possible to very rapidly get a comprehension of possible crosslinks that are present in a crosslinked sample. After protein crosslinking the tryptic digest is subjected to LC-fractionation in order to reduce ion suppression and promote the low-abundant crosslinked peptides, and MS-data acquisition is performed as a first step to screen for potential crosslinked peptides. The software FINDX merges the peak list files for all the LC-separated fractions and recognizes candidate crosslinked peptides as isotope-labeled doublets. Data on the peak mass difference between the isotope doublet peaks, and their intensities, can be utilized by FINDX to reduce the number of false positives. In a second step MSMS-spectra are acquired for crosslink validation, using an inclusion list that is generated based on the information from the MS-step. This acquisition step is also comparatively rapid, since only a restricted number of precursors are selected for MSMS. The LC-MALDI TOF/TOF workflow here described, especially in combination with a ^14^N/^15^N mixed isotope strategy, is useful to investigate crosslinking within and between subunits in protein oligomers.

## Results and Discussion

### 1. Decreasing False Positives by Search Constraints in FINDX Considering the Isotope Doublet Peaks in the MS-spectra

For a workflow based on MS-data analysis of single proteins crosslinked with the lysine-specific crosslinkers DTSSP and BS^3^ we started to develop the software FINDX to assist the LC-MALDI-TOFTOF-based data analysis and bring down the number of MSMS-spectra needed to be acquired for crosslink validation [13]. The FINDX procedure allowed for more efficient use of instrument time and the restricted number of MSMS-spectra benefits the quality of the MSMS data.

In this work, FINDX was further developed to permit crosslink identification based on MS-data also with more complex crosslinked samples, containing more than one protein. With the increased complexity of the tryptic digest, a shallower gradient and an increased number of collected fractions must be used during separation by nano-LC, in order to reduce ion suppression. For example, it was recently shown that 13682 peptides were identified using a 480 min gradient, compared to only 5806 peptides using a 140 min gradient [14]. If one increases the number of fractions from 64 to 192 in 192 wells on a MALDI target plate, fewer other peptides will elute into the same well where the low-abundant crosslinked peptide is eluting. This will reduce the ion suppression and increase the S/N of crosslinked peptides in the MS spectrum, and accordingly, the corresponding MSMS-spectra will be better.

**Figure 1:**
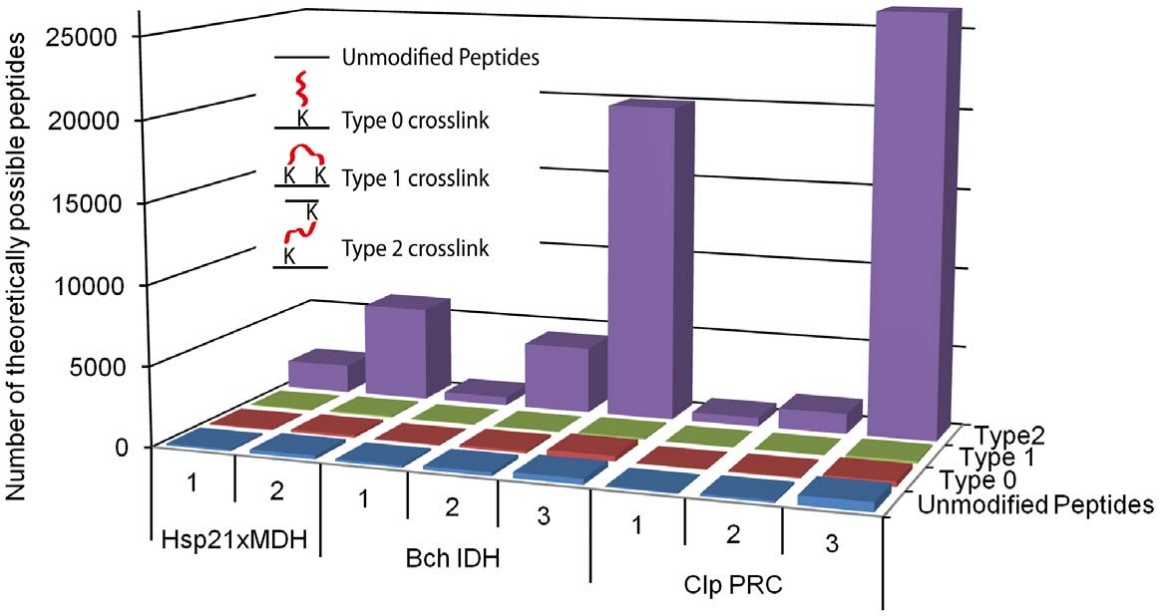
The number of possible crosslinked peptides increasing with sequence length. The number of theoretically possible peak masses of tryptic unmodified peptides and of crosslinked peptides between 600 and 5000 Da was calculated using the software GPMAW. Data shown from left to right for a single chaperone protein here investigated, Hsp21 (UniProtKB P31170; first 44 amino acids replaced with start methionine, sequence length 184), and a chaperone model substrate protein used in ongoing crosslinking experiments MDH, (P00346, sequence length 338), and two AAA protein complexes. The AAA protein magnesium chelatase Bch IDH complex from *Rhodobacter capsulatus* is composed of subunit I (P26239, and C-terminal hexa-His, sequence length 356, 10 lysines), subunit D (P26175, and N-terminal hexa-His, sequence length 567, 15 lysines), and subunit H (P26162, and N-terminal hexa-His, sequence length 1195, 44 lysines). The AAA protein Clp-protease from *Synechococcus sp.* is composed of subunit P (Q9L4P3, and C-terminal hexa-His tag, sequence length 205, 9 lysines), subunit R (Q9L4P4, sequence length 228, 7 lysines), and subunit C (Q55023, sequence length 824, 52 lysines).

However, with a larger number of LC-fractions the total number of detected peaks to consider increases, and the number of false positive matches also increases. The false positive matches are peak masses that by chance match to masses of theoretically possible crosslinked peptides, which especially in a complex sample is a very large number. The number of theoretically possible masses of crosslinked peptides increases quadratically with the number of subunits in the protein complex, whereas the number of theoretically possible unmodified peptide, type 0 crosslink, and type 1 crosslink peak masses increases only linearly, as exemplified in Fig. 1 for the protein complexes analyzed here. To reduce the number of false positive matches in the MS-data from crosslinked protein complexes, the search constraints in FINDX can optionally be made stricter by taking into consideration the mass difference between the isotope doublet peaks and the intensities of the isotope doublet peaks as described below.

### 1.1 Search Constraint in FINDX Considering the Mass Difference between the Isotope Doublet Peaks

The mass accuracy in a MALDI-MS spectrum is strongly dependent on internal calibration, and the accuracy of the mass difference between the isotope doublet peaks may therefore be considerably better than the mass accuracy of the spectrum. This value for the accuracy of the relative mass difference between the isotope doublet peaks is therefore fixed in FINDX, such that candidate crosslinks are only considered if the peak mass difference between the hydrogenated form (H12) and the deuterated form (D12) is 12.0757 Da plus or minus maximally 7 ppm (very restrictive filter). Optionally, this value can be fixed to 20 ppm (less restrictive filter), or not fixed, it then varies with the tolerance setting in the search. The search constraints imposed by the fixed values for the isotope doublet mass difference accuracy is valuable to reduce false positives in case of complex samples, and in case of MS-spectra with only default calibration where the search tolerance may need to be increased to above 20 ppm. Failed internal calibration usually occurs in a few wells of the 192 wells on the target plate, often in the most interesting wells where amount of eluted peptides is high, hence causing ion suppression of the internal calibrant peptides.

### 1.2 Search Constraint in FINDX Considering the Intensities of the Isotope Doublet Peaks

The ratio of the two peak intensities of the isotope doublet peaks should in principle be 1∶1, unless neighboring peaks suppress or overlay one of the isotope doublet peaks. When using the BS^3^ crosslinker, two peaks appearing in the peak list with a 12 Da mass difference that are *not* true isotope doublet peaks will not show such a 1∶1 intensity ratio, and are indeed easily recognized as false positives by manual inspection of the MS-spectra.

**Figure 2:**
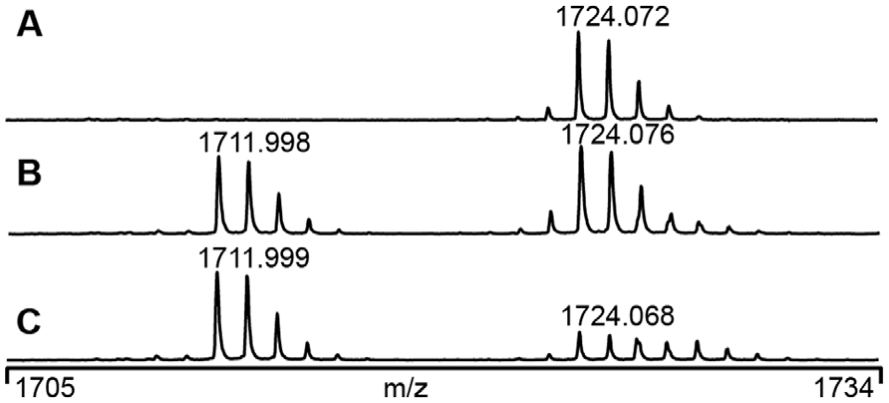
Isotope doublet peaks eluting with D12 before H12. MS-spectra showing typical elution pattern for a crosslinked peptide in three consecutive LC-fractions (A, B, C) in a 192-well LC-separation. This crosslinked peptide has a theoretical mass MH^+^1712/1724 Da for H12/D12, and corresponds to the crosslink between T_172_KVER_176_ and K_177_VIDVQIQ_184_ (Table 1).

To filter away these false positives without manual inspection of spectra, FINDX is therefore designed to consider also the intensity information in the peak list file, with the option to impose the requirement of a 1∶1 ratio between the intensities of isotope doublet peaks as an efficient way to reduce the number of false positives. Yet, in order not to miss *true* positives, deviations from a 1∶1 ratio must be allowed. This is due to the isotope elution difference, previously known for ICAT [15], such that the D12 peak appears earlier than the H12 peak as illustrated in Fig. 2. This isotope elution difference effect becomes more pronounced with a larger number of LC-separated fractions (e.g. 192 wells versus 64 wells). This typical peak pattern in the MS-spectra, actually a very good hallmark of a true crosslinked peptide, means that if the crosslinked peptide elutes in more than one LC-fraction, none of the MS-spectra may show a 1∶1-ratio. Therefore instead of imposing a strict 1∶1 ratio requirement, the allowed intensity ratio between the two isotope doublet peaks can be specified in FINDX. This adjustable intensity parameter is calculated as the difference between the isotope doublet intensities divided by the average isotope doublet intensity, i.e. the situations where the intensity of one of the peaks is for example 82%, 80%, 74% and 50% of the other, are corresponding to the settings 0.2, 0.22, 0.3 and 0.67 of the intensity parameter.

**Table 1:** Crosslinks detected by FINDX within a single protein using the crosslinker BS^3^.

Theoretical[MH]^+^ (Da)	Matched sequences	aCrosslinked amino acid residues	bDistance Cα-Cα (Å)in dimer	cMixed ^14^N/^15^N pattern
1272.75	D_154_KIK_157_, T_172_KVER_176_	K155–K173	14 (m)	*intra*
1446.74	M_1_QDQR_5_, T_172_KVER_176_	M1–K173	Unstr.	*inter*
1470.89	I_156_KAELK_161_, T_172_KVER_176_	K157–K173	18 (m)	*intra*
1491.67	M_1_QDQR_5_, M_1_QDQR_5_	M1–M1	Unstr.	-
1515.82	M_1_QDQR_5_, I_156_KAELK_161_	M1–K157	Unstr.	*inter*
1712.00	T_172_KVER_176_, K_177_VIDVQIQ_184_	K173–K177	12 (m)	*intra*
1756.93	M_1_QDQR_5_, K_177_VIDVQIQ_184_	M1–K177	Unstr.	*inter*
2014.93	M_1_QDQR_5_, E_90_EEHEIKMR_98_	M1–K96	Unstr.	-
2092.06	F_99_DMPGLSKEDVK_110_, G_122_EQKK_126_	K106–K125	13 (m)	-
2095.90	M_1_QDQR_5_, K_126_EDSDDSWSGR_136_	M1–K126	Unstr.	*inter*
2141.14	M_1_QDQR_5_, V_174_ERKVIDVQIQ_184_	M1–K177	Unstr.	*intra*
2180.04	M_1_QDQR_5_, F_99_DMPGLSKEDVK_110_	M1–K107	Unstr.	*inter*
2269.32	G_122_EQKK_126_, A_158_ELKNGVLFITIPK_171_	K125–K161	17 (m)	-
2379.21	G_122_EQKK_126_, M_97_RFDMPGLSKEDVK_110_	K125–K106	13 (m)	-
2385.17	M_1_QDQR_5_, G_19_NQGSSVEKRPQQR_32_	M1–K27	Unstr.	*inter*
2393.24	A_84_PWDIKEEEHEIK_96_, T_172_KVER_176_	K89–K173	19 (m)	-
2438.17	M_1_QDQR_5_, A_84_PWDIKEEEHEIK_96_	M1–K89	Unstr.	-
2440.37	I_111_SVEDNVLVIKGEQK_125_, T_172_KVER_176_	K121–K173	28 (x)	*inter*
2485.30	M_1_QDQR_5_, I_111_SVEDNVLVIKGEQK_125_	M1–K121	Unstr.	*inter*
2619.16	E_90_EEHEIKMR_98_, K_126_EDSDDSWSGR_136_	K96–K126	11 (d)	-
3042.40	A_84_PWDIKEEEHEIK_96_, K_126_EDSDDSWSGR_136_	K89–K126	12 (d)	*inter*
3089.53	I_111_SVEDNVLVIKGEQK_125_, K_126_EDSDDSWSGR_136_	K121–K126	20 (m)	-
3128.58	E_6_NSIDVVQQGQQKGNQGSSVEK_27_, T_172_KVER_176_	K18–K173	Unstr.	-
3173.51	M_1_QDQR_5_, E_6_NSIDVVQQGQQKGNQGSSVEK_27_	M1–K18	Unstr.	*intra*

Crosslinking was performed with Hsp21, an oligomeric chaperone small heat shock protein [19], at a protein concentration of 50 µM. The data shown in the table is the sum of crosslinks observed in three independent experiments with crosslinker to protein ratios of 1∶1, 10∶1 and 50∶1. Only crosslinks of type 2 are shown (not type 0 and 1). Crosslinks were detected with LC-MALDI-TOFTOF by first using the MS-data for screening in FINDX, and subsequent validation of all crosslinks by MSMS.

a Crosslinked lysine residues, designated with amino acid number in sequence (M1 refers to crosslinking with the primary amine in the N-terminal residue, methionine).

b The distances between the crosslinked lysine residues in the three-dimensional structure model of Hsp21 [19] are below 20 Å and the cross-links reconcilable with the 3D-structure, m  =  within monomer, d  =  within dimer, (x)  =  one distance was not <20 Å, presumably a crosslink between subunits or oligomers as suggested by its inter-monomeric status in the right-most column, Unstr.  =  crosslinks involving either M1 or lysine residues in the unstructured N-terminal domain (residues 1–81 in sequence) for which the distance cannot be determined.

c *intra* and *inter*  =  intra- and inter-monomeric, -  =  crosslink not examined by ^14^N/^15^N mixed isotope crosslinking as described in Fig. 4.

### 2. The Crosslinks Detected by FINDX Map Well into Protein Structure

**Figure 3:**
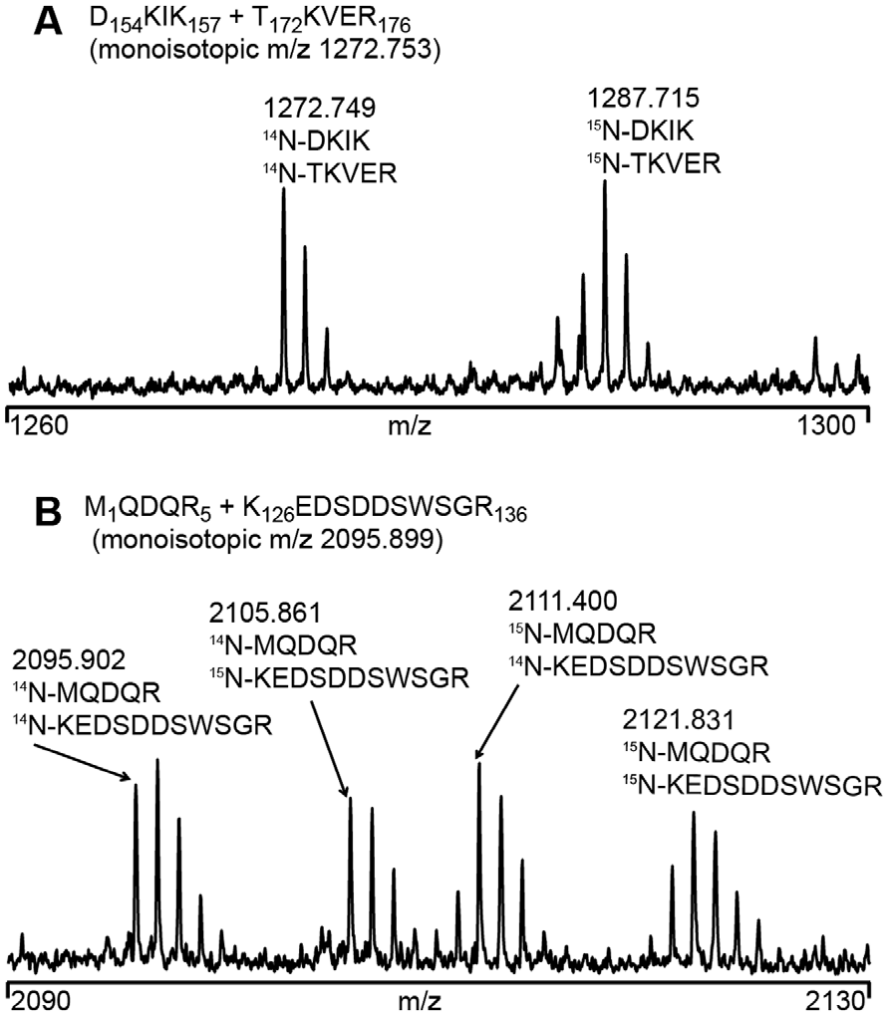
Crosslinks detected by FINDX map into the protein structure. Crosslinks detected by FINDX and validated by MSMS are listed in Table 1 and here mapped into the structure of the Hsp21 protein between the Cα atoms of two lysines. Assuming a maximum distance between the two Cα atoms of crosslinkable lysines of 20 Å (12+8 Å  =  length of the BS3 crosslinker and 2 x the lysine side chain), all identified intra-dimeric crosslinks are reconcilable with the dimer structure. The Hsp21 protein is composed of six dimers [16] but for clarity only one dimer is presented with subunit A (to the left, dark grey) and subunit B (to the right, lighter grey). Two crosslinks that are inter-monomeric involve the lysine residue K126, which is located in the flexible loop with strand β6, that stabilizes the dimers via strand exchange between the two monomers [24]. The image of the Hsp21 structure model [19] was prepared with PyMOL (http://www.pymol.org).

Lysine-lysine crosslinks that were detected by screening the MS-spectra with FINDX and then validated by MSMS are listed in Table 1 for a single protein. This protein, a plant small heat shock protein, Hsp21, and its homologue, human αB-crystallin, are both oligomeric chaperone proteins, the former is a dodecamer based on dimers [16] and the latter a polydisperse ensemble of up to 48 subunits, also based on dimers [17]. We previously showed for these two proteins that all the crosslinked peptides were within crosslinking distance (<20Å) and mapped well into the dimer structure [13]. Since most of the crosslinks were readily explained by distances <20 Å within the monomers we assumed that most of the crosslinks were indeed intra-monomeric. The detected crosslinks were mapped into the structure, as shown in Fig. 3.

### 3. To Distinguish Intra-monomeric and Inter-monomeric Crosslinks

**Figure 4:**
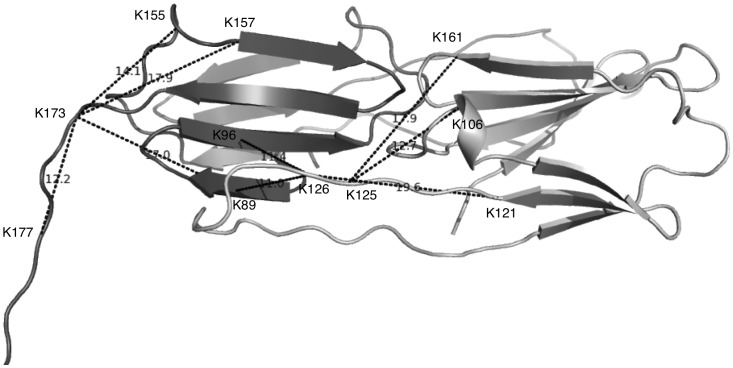
Intra- and inter-monomeric crosslinking distinguished by mixed isotope labeling. MS-spectra obtained by crosslinking of ^14^N/^15^N oligomers of Hsp21 showing (A) a crosslink formed within the monomers resulting in two peaks with ^14^N-^14^N and ^15^N-^15^N (designated as intra-monomeric (*intra*) in Table 1) and (B) a crosslink formed between the monomers resulting in a four peak pattern with ^14^N-^14^N, ^14^N-^15^N, ^15^N-^14^N and ^15^N-^15^N (designated as inter-monomeric (*inter*) in Table 1). The inter-monomeric crosslinks can originate from crosslinking within or between the oligomers. The two-peak pattern with ^14^N-^14^N and ^15^N-^15^N could in principle also originate from inter-monomeric intra-dimeric crosslinking since stable dimers may be the exchanging subunits [25], [26].

In order to determine unequivocally whether the crosslinks are intra-monomeric or inter-monomeric, mixed isotope crosslinking [18] was conducted with a 1∶1 mixture of unlabeled ^14^N and ^15^N-labeled Hsp21 oligomers (Fig. 4). The mixture was allowed to equilibrate by subunit exchange until ^14^N/^15^N-labeled oligomers had formed, and then crosslinking was performed. Unlabeled crosslinker was used in order to facilitate the spectrum interpretation, and the known peak masses of the crosslinks, previously identified using the isotope-labeled crosslinker (Table 1), were taken as starting point for inspection of the pattern displayed in the MS-spectra. Either only two peaks were visible (Fig. 4A, ^14^N/^14^N and ^15^N/^15^N, indicative of crosslinking within the monomers, or four peaks (Fig. 4B, ^14^N/^14^N and ^15^N/^15^N and combinations between them, indicative of crosslinking between monomers). These data, listed in the right-most column in Table 1, confirmed that the majority of the detected crosslinks that are mapped into the dimer structure, as shown in Fig. 3, are intra-monomeric. Two crosslinks are intra-dimeric and only few are intra-dodecameric, or possibly inter-dodecameric.

Nearly half of the detected crosslinks involve M_1_QDQR_5_ and there are several reasons for this. Firstly, the peptide M_1_QDQR_5_ is R-terminated and short, two good properties for becoming ionized and getting detected, secondly, the N-terminal arm is very flexible, and thirdly, the pKa-value of the N-terminal amine is lower compared to lysine side-chain amines making this peptide very reactive. The mixed isotope crosslinking data in Table 1 reveal that the M_1_QDQR_5_-crosslinks are predominantly inter-monomeric. Many are probably intra-dodecameric, holding the Hsp21 dodecamers together, and indeed, crosslinked N-terminal arms appear visible as extra density in the interior of crosslinked dodecamers in image reconstructions generated by negative stain single particle EM [19]. Probably, some inter-monomeric M_1_QDQR_5_-crosslinks are also inter-dodecameric. One example is the crosslink with MH^+^ = 2440.3, I_111_SVEDNVLVIKGEQK_125_, T_172_KVER_176_, with two lysines that are located on either side of a monomer. The mixed isotope crosslinking data show that it is inter-monomeric, unlikely to be intra-dodecameric because all K121–K173 distances within a dodecamer are >30 Å, so it is presumably inter-dodecameric.

**Figure 5:**
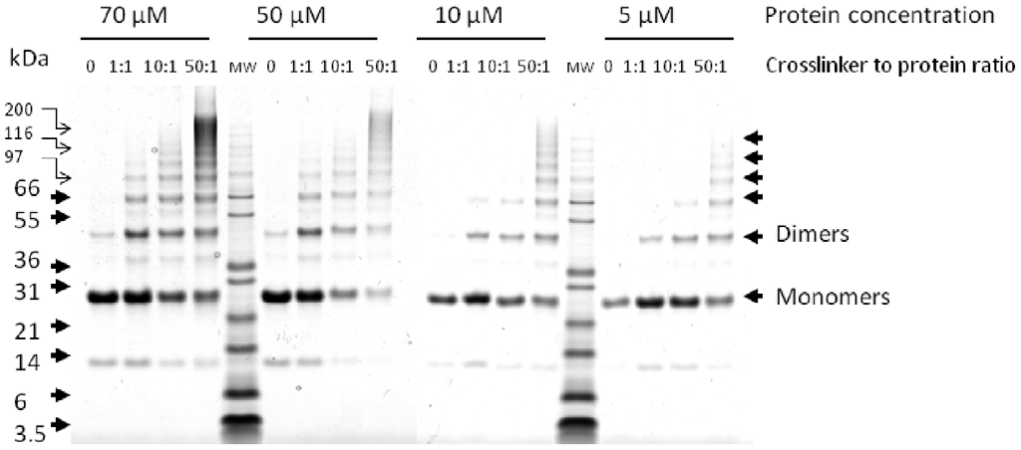
Crosslinking with varying protein concentration and protein-to-crosslinker-ratio. Crosslinking of the dodecameric Hsp21 protein at varying protein concentrations (5, 10, 50 and 70 µM) and crosslinker to protein molar ratio (1∶1, 1∶10, 1∶50) was evaluated by SDS-PAGE. To each lane, 1.2 µg crosslinked protein was loaded (except for the 70 µM samples where 2.4 µg was loaded) and the gel was stained with CBB.

Crosslinking performed at different protein concentrations and different crosslinker to protein ratios yields different proportions between crosslinked monomers, dimers and other oligomeric forms, as visualized by SDS-PAGE (Fig. 5). The higher the crosslinker to protein ratio the more crosslinks were detected. Samples excised from the monomer band only contained so called dead-end (type 0) crosslinks, and samples from the other bands contained fewer type 2 crosslinks than the unfractionated crosslinked Hsp21. The best yield of structurally informative crosslinks of type 2 was obtained not from samples excised from gel but from in-solution digests of unseparated crosslinked protein.

Altogether these data show that the crosslinked peptides detected by FINDX based on MS-data and validated by MSMS are reconcilable with structure and represent true spatial constraints, and that, apart from the crosslinks with the flexible and reactive N-terminal, most of the crosslinks detected in this oligomeric protein are intra-monomeric.

### 4. Using FINDX MS to Detect Crosslinks within Multisubunit Protein Complexes

**Figure 6:**
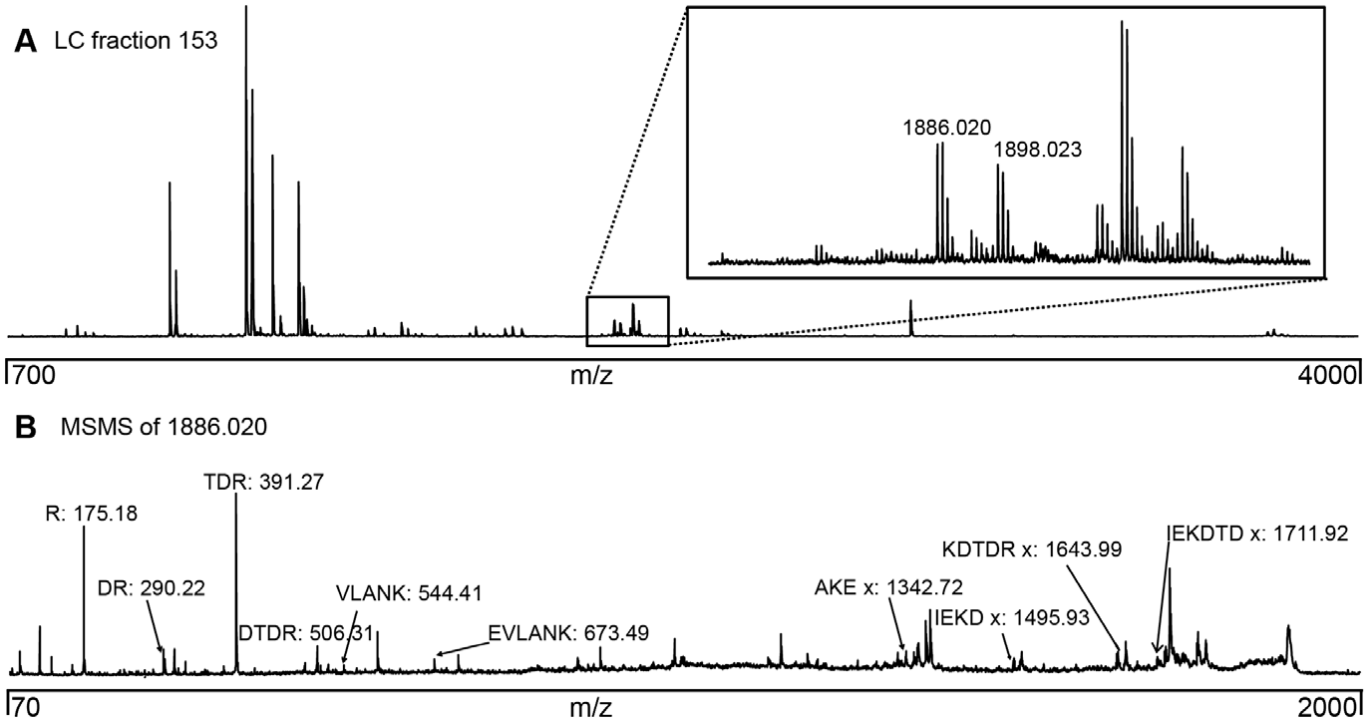
Mass spectra for crosslinks identified in protein multisubunit complexes. The Clp-protease multisubunit protein complex was crosslinked and the tryptic digest was separated into 192 fractions on the MALDI-target plate. The MS-data were analyzed in FINDX with search restrictions on the isotope doublet peak mass difference accuracy (7 ppm) and intensities (intensity parameter set to 0.22) and matched against >25000 theoretically possible peak masses. Only a few (12) peaks were suggested to be crosslinks, and subjected to MSMS. (A) MS-spectrum showing typical isotope doublet peaks recognized by FINDX. This crosslinked peptide has a theoretical mass MH^+^1886/1898 Da for H12/D12, and corresponds to the crosslink between subunit P_3_ peptide I_169_EKDTDR_175_ and subunit R peptide A_164_KEVLANK_171_ and was detected only in 1 out of 192 wells on the MALDI target plate. (B) MSMS-spectrum acquired for crosslink validation. All the fragments containing the crosslinker (AKEx, IEKDx, KDTDRx, EKDTDx) appear as doublet peaks, further confirming the identification.

**Figure 7:**
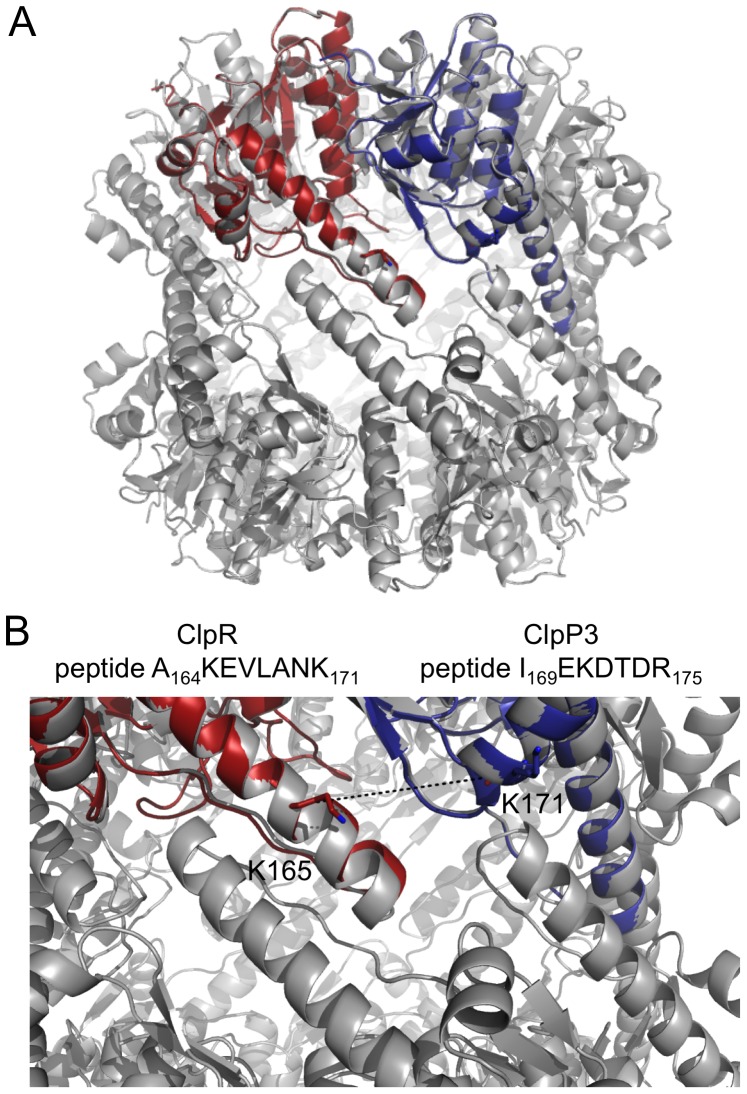
The P_3_-R crosslink in the proposed subunit organization of the ClpP_3_/R complex. The identified crosslink between peptide A_164_KEVLANK_171_ of subunit R and peptide I_169_EKDTDR_175_ of subunit P_3_ fits the proposed structure model of the Clp proteolytic core complex. A: *E. coli* ClpP shown in gray (PDB ID 1TYF), with one R (in red) and one P3 (in blue) *Synechococcus* subunit superimposed on two *E. coli* ClpP subunits. B: The distance between the Cα atoms of the crosslinked lysine residues is 19 Å in this model, which is compatible with the length of the crosslinker. The figures were prepared with PyMOL (www.pymol.org). The homology models of ClpR (UniProtKB Q9L4P4) and ClpP3 (UniProtKB Q9L4P3) were downloaded from ‘The Protein Model Portal’ (www.proteinmodelportal.org), with the models based on templates 1tyfC (residues 24–217) and 1tyfA (residues15–197). They were superimposed onto chains C and D of *E. coli* ClpP with the ‘magic fit’ function in Swiss-PdbViewer [27].

With the stricter search constraints in FINDX the number of false positives was reduced sufficiently to permit identification of crosslinks by MS-data also in multisubunit protein complexes. For the Clp-complex, an intricate and large 546 kDa AAA-type protease in plant chloroplasts [20] with three different subunits, two (subunits P_3_ and R) in a proteolytic core with two heptameric rings, and one (subunit C) in a ATP-hydrolyzing hexameric ring, the number of theoretically possible masses of crosslinked peptides is >26000, as outlined in Fig. 1. After crosslinking and acquisition of MS-data the number of suggested candidate crosslinks to be investigated by MSMS was 450 with no isotope doublet restriction at all, 34 with a fixed value of 20 ppm for the mass error of the difference between the isotope doublet peaks, 18 with a fixed value of 7 ppm, and 12 with the additional requirement of the 1∶1 intensity ratio between the isotope doublet peaks with the intensity parameter set to 0.22. Thus, the number of peaks to be further fragmented by MSMS for crosslink validation was reduced to only 12. As exemplified in Fig. 6A for one of these peaks, the MS-spectrum showed isotope doublet peaks at 1886.0 and 1898.0 Da, matching the crosslink between the P_3_-subunit (I_169_EKDTDR_175_) and the R-subunit (A_164_KEVLANK_171_). The MSMS-spectrum confirmed the identity of this inter-subunit crosslink (Fig. 6B), which indeed also fits the proposed subunit organization of the two heptameric rings of the ClpP_3_/R proteolytic core (Fig. 7). Another complex AAA-type protein, the 958 kDa magnesium chelatase from *Rhodobacter capsulatus*, also yielding a complex peptide mixture and a large search space of theoretically possible crosslinks (Fig. 1), was crosslinked and several inter-subunit crosslinks between the BchD and BchH subunits were detected (Peterson Wulff et al, in preparation), allowing entirely new conclusions concerning how the subunits are organized. Thus even in more complex systems as these multisubunit protein complexes it is possible, with stricter search constraints in FINDX, to screen for and select possible crosslinks based on MS-data.

### 5. FINDX and Other Types of Software for Lysine-specific Crosslinkers

Today software exist that works with the lysine-specific crosslinker BS^3^ by which data from online LC-MSMS can be analyzed. The well-known xQuest algorithm [21] works with isotopically coded lysine-specific crosslinkers, and is capable of identifying cross-linked peptides from very complex samples and large sequence databases. Samples containing crosslinked peptides are subjected to online LC-MS and peptide masses screened for isotopic pairs, for which MSMS-spectra are recorded and analyzed according to the absence or presence of an isotopic shift between peaks in the fragment ion spectrum. The light and heavy form of isotopic peptide pairs are subjected to separate MSMS fragmentation, and fragment ions that are present in both are used for protein identification. Fragment ions present in either light- or heavy spectra are used to identify crosslinks after matching into a small combinatorial database with peptides from the identified proteins. CrossWork [11], an even more recent software, uses non-isotopically encoded lysine-specific crosslinkers and data-dependent MS/MS scans performed on the most intense multiply charged ions after an initial MS-scan on a platform such as a nano-LC LTQ-Orbitrap XL mass spectrometer. CrossWork is based on the observation that the larger peptide in a cross-link generally generates more high intensity fragments than the shorter in MS/MS. By searching the most intense peaks, the identity of one of the peptides is established. The shorter, more poorly fragmenting peptide is then recognized first by mass only, and then validated by the MS/MS fragments. This approach reduces the search to that of comparing the list of cross-linkable peptides twice per scan, rather than comparing the same scan to a full mass-list of every possible cross-link. Like xQuest, CrossWork has an advanced scoring algorithm (CWscore), which scores the overall quality of the scan by taking a number of features (Fscores) into account. Using CrossWork, 4455 MSMS scans representing 291 linear peptides and 700 scans representing 86 different crosslinks were collected and mapped into the known structure, and a 70 kDa protein was structurally characterized using 17 samples crosslinked with BS3 with a total of 47000 MSMS scans.

Compared to the above-mentioned MSMS-based softwares, FINDX by itself may not represent a significant advance in analyzing isotopically coded lysine-specific crosslinkers. Yet the LC-MALDI TOF/TOF workflow here described, especially in combination with the ^14^N/^15^N mixed isotope strategy, is a straightforward and working solution to investigate crosslinking within and between subunits in protein oligomers. Furthermore, the overall workflow and FINDX restricts the amount of MSMS-acquisition and directs it to crosslinked peptides, and especially to the Type 2 crosslinks. In the datasets we have investigated, unmodified peptides are undoubtedly the most abundant, closely followed by peptides with a dead-end crosslink modification (type 0) and after this, intra-peptide crosslinks (type 1) follow in their abundance. Thereafter, inter-peptide crosslinks (type 2) within the same subunit (*intra-monomeric*) are abundant enough to be fairly easy to detect, and next in abundance are inter-peptide crosslinks (type 2) between two subunits (*inter-monomeric*), within a protein oligomer, or within a stable protein complex as in the example shown in Figs. 6 and 7. Finally, inter-peptide crosslinks (type 2) between two transiently interacting proteins are by far the least abundant. Compared to existing methods FINDX has some advantage in the MS-based peptide sorting, such that MSMS is directed to and focused on crosslinks of Type 2, which are of highest biological interest but often lowest in abundance.

### 6. Concluding Remarks

Lysine-specific crosslinking can be evaluated using off-line LC-MALDI-TOF/TOF mass spectrometry in a two-step work-flow that permits fast detection of possible crosslinks based on MS-data, and subsequent validation of crosslinked peptides by a limited number of MSMS-spectra. Acquisition and analysis of the MS-data by FINDX is rapidly achieved within an hour on the user-friendly MALDI platform. When limited to only a few selected peaks, high-quality MSMS-data can subsequently be acquired for validation, either with the same sample still in place on the MALDI target plate, or with a new aliquot of the sample on another platform. The LC-MALDI TOF/TOF workflow here described is useful in combination with a ^14^N/^15^N mixed isotope strategy to distinguish crosslinking within and between subunits in protein oligomers. The program FINDX is available on request via e-mail to findxlink@gmail.com or through the website http://findxlinks.blogspot.com/.

## Materials and Methods

### 1. Proteins and Reagents

Recombinantly expressed Hsp21 from *Arabidopsis thaliana* (sequence as in UniProtKB P31170 with the first 44 amino acids replaced by a start methionine) was obtained as previously described [16]. Hsp21 protein labeled with ^15^N was obtained by growing the bacterial host in minimal medium containing ^15^N-NH_4_Cl as nitrogen source and was purified as the unlabeled protein. The *Synechococcus* ClpCP_3_R AAA-type protein complex was obtained as described previously according to [20]. Proteins were desalted and buffer exchanged into crosslinking buffer (50 mM HEPES pH 8.0, 150 mM NaCl, 5 mM MgCl_2_) using disposable PD-10 protein desalting columns (GE Healthcare, Little Chalfont, UK). Protein concentrations were determined with the Bradford assay [22] using *Bos taurus* (bovine) serum albumin as a standard. The isotope-labeled crosslinking reagent bis(sulfosuccinimidyl) suberate (BS^3^), consisting of a 1∶1 molar ratio mixture of BS^3^-H12 and BS^3^-D12, was obtained from Creative Molecules Inc. (Victoria, Canada). Unlabeled crosslinker BS^3^ was obtained from Pierce (Thermo Fischer Scientific inc., Rockford, US).

### 2. Chemical Crosslinking

The protein concentration was 50 µM, or else as indicated. The crosslinker was dissolved in distilled water to a concentration of 30 mM immediately before use. Sample aliquots of 20 µl were incubated with a 1∶1 mixture of H12/D12 isotopically coded BS^3^ (final concentration 3 mM, or less where indicated) at 25°C. After 15 minutes the crosslinking reaction was quenched by adding 1 M tris-(hydroxymethyl)-aminomethane (Tris) to a final concentration of 20 mM. To remove excess reagent and Tris and to concentrate the proteins, the samples were precipitated with freeze-cold acetone.

### 3. Mixed Isotope Crosslinking

For mixed isotope crosslinking, unlabeled (^14^N) and ^15^N-labeled Hsp21 protein were mixed 1∶1, incubated for 1 hour at 37°C, and subsequently crosslinked with non-isotopically coded BS^3^ and analyzed as described for the non-^15^N-labeled samples. The masses of already detected and validated crosslinks were used to calculate the corresponding masses of crosslinks with different proportions of unlabeled and ^15^N-labeled protein. Upon evaluation of MS-spectra crosslinks were assigned as intra-monomeric (‘*intra*’) or inter-monomeric (‘*inter*’) as follows: If only ^14^N-^14^N and ^15^N-^15^-N (should be about 1∶1 intensity) were present: *intra*. If all 4 variants were present: *inter*. The 4 variants situation can be split into two situations: 1. ^14^N-^14^N and ^14^N-^15^-N and ^15^N-^14^N and ^15^N-^15^-N intensities  = 1∶1:1∶1 and 2. ^14^N-^14^N and ^14^N-^15^-N and ^15^N-^14^N and ^15^N-^15^-N intensities  = 1: x, for example x = 0.5, with the former representing a situation with only inter-monomeric crosslinking, the latter a mixture of both inter-monomeric and intra-monomeric crosslinking. In case of a mixture of both inter-monomeric and intra-monomeric crosslinking, the crosslink was actually designated *inter*-monomeric, because the intra-monomeric signal most likely does not represent real intra-monomeric crosslinks, but appears because the differently labeled oligomers have not exchanged subunits to the equilibrium situation.

### 5. Analysis of Crosslinked Protein by SDS-PAGE

To evaluate the results of the crosslinking reactions, aliquots of the crosslinked samples were withdrawn and proteins separated by denaturing gel electrophoresis using precast 4–12% NuPAGE® SDS-PAGE Bis-Tris Gels (Life Technologies Europe BV, Stockholm, Sweden) according to the manufacturer’s instructions. Samples were solubilized using LDS sample buffer (4x), heated for 10 min at 90°C and loaded on the gel that was run at 200 V for 40 min with MES running buffer. For staining the protein bands, the gel was washed three times with ddH_2_O (30 sec in the microwave oven, followed by 5 min shaking) and stained with Coomassie Brilliant Blue G-250 (60 mg in 1 L ddH_2_O and 3 mL concentrated HCl) according to the Quick-stain colloidal CBB protocol (http://www.jove.com) and scanned on an Image Scanner III (GE Healthcare LifeSciences, Uppsala, Sweden).

### 6. Trypsin Digestion

The protein pellets from acetone-precipitation were carefully re-dissolved in 20 µl 25 mM NH_4_HCO_3_ pH 7.8. The samples were digested with sequencing-grade modified trypsin (Promega, Madison, WI, USA) at 37°C at a protease:protein (w/w) ratio of 1∶100 for 1 h, followed by a ratio of 1∶50 over-night. All samples were acidified by adding 2 µl 10% trifluoroacetic acid (TFA) and stored at −20°C until further analysis.

### 7. Reversed Phase Liquid Chromatography

The samples were separated by reversed phase liquid chromatography using an 1100 Series Nanoflow LC system (Agilent Technologies, Waldbronn, Germany). The mobile phases used for separation were composed of A: 1% (v/v) acetonitrile and 0.1% (v/v) TFA, and B: 90% (v/v) acetonitrile and 0.1% (v/v) TFA. For each sample, 2, 8 or 20 µl was injected, which was estimated to contain approximately 50, 200 or 500 pmol peptides. Samples were loaded onto a Zorbax 300SB-C18 0.3 mm pre-column (Agilent Technologies) in buffer A at a flow rate of 0.040 ml/min, delivered by the isocratic pump. By switching the micro 6-port/2-position module, the nano-pump delivering a linear gradient from 0 to 100% buffer B (flow rate 1.4 µl/min) was then connected to the pre-column to move the sample onwards to the separation column, a PepSwift Monolithic Capillary Column (200 µm i.d. x 5 cm) (Dionex, Amsterdam, the Netherlands). Before starting to collect fractions for a sample, 2 µl was injected to saturate the column and eluted into waste. Peptides eluted between about 5 and 45% buffer B, and were collected in 64 (usually for single protein samples) or 192 fractions (in case of complex protein samples) on the MALDI target plate.

### 8. MALDI-TOF/TOF Mass Spectrometry

Matrix solution consisting of 5 mg/ml α-cyano-4-hydroxy cinnamic acid, 50% acetonitrile, 0.1% TFA, 25 mM citric acid, and standard peptides (Angiotensin II, m/z 1046.541 Da; Neurotensin, m/z 1672.918 Da and ACTH 18-39, m/z 2465.199 Da) for internal calibration, was manually applied to the dried peptide fractions and allowed to dry. Mass spectrometric data were acquired using a 4700 Proteomics Analyzer (Applied Biosystems/MDS SCIEX, USA) in the Positive Reflector mode. The spectra were internally calibrated using the standard peptides. After MS-data analysis by FINDX (see below), inclusion lists with peaks suggested to be crosslinked peptides (the light isotope peak masses used in the inclusion list) were used for selection of crosslink precursors for MSMS fragmentation. MSMS spectra were recorded in the MS-MS 1 kV Positive mode with 3000 laser shots per spectrum, or manually with various settings to optimize the output.

### 9. Data Analysis by FINDX

The program FINDX was written in the Python 2.7 programming language and can be used to analyze MS and MSMS data from crosslinking experiments with the crosslinking reagent.

BS^3^ (an earlier version is also compatible with the crosslinker DTSSP). When using isotope-labeled forms of crosslinking reagent, only MS peaks that display an isotope doublet of peaks are considered as possible crosslinked peptides.

In MS-mode, the program first filters the data from the MS spectra from the LC fractions by removing masses not having one peak mass in presumed crosslinker hydrogenated form (H12) and a corresponding peak mass in presumed crosslinker deuterated form (D12). Subsequently, the filtered peaklists are merged into one peaklist file. The masses in the filtered peaklist are then compared against a theoretical list including all dead-end, intra-peptide, and inter-peptide crosslinks, as well as optionally any of those with one extra dead-end cross-linker. The definition of a dead-end crosslink is that one end of the cross-linker has reacted with an amine from the protein, and the other end has either been hydrolyzed or has reacted with Tris, which was used to quench the reaction. The modification by BS^3^ resulting in an inter-peptide or dead-end crosslink corresponded to a mass difference of 138.07 Da and 156.08 Da, respectively. The MS precursor tolerance, the enzyme used, the number of missed cleavages, the allowance of methionine oxidations can all be specified prior to analysis. Also, settings can be chosen for the accuracy of the mass difference between the isotope doublet peaks, and for the allowed intensity ratio between the two isotope doublet peaks (difference between the isotope doublet intensities divided by the average isotope doublet intensity), such that the settings 0.2, 0.22 and 0.3 correspond to the situations where one of the peaks is 82%, 80%, and 74% of the other, respectively. The inclusion list generated by FINDX in MS mode is based on the light isotope peak masses, and used to acquire MSMS data. Due to the broad m/z window in the timed ion selector, both light and heavy peaks from isotopic pair will be fragmented and doublet peaks are frequently observed in the MSMS, further supporting that the precursor ion is indeed a crosslinked peptide.

In MSMS mode, the program first merges the data from the MSMS-spectra from all the LC fractions into one.mgf file (.mgf, Mascot Generic Format). The precursor masses are matched and the fragment experimental masses are subsequently matched to the theoretical fragments. During the development of FINDX, data were also analyzed using the program GPMAW (Lighthouse data, Odense, Denmark [23]). The program FINDX is available on request via e-mail: findxlink@gmail.com.
