# Correction: Detection of Crosslinks within and between Proteins by LC-MALDI-TOFTOF and the Software FINDX to Reduce the MSMS-Data to Acquire for Validation

**DOI:** 10.1371/annotation/6a624d85-7565-4614-9c8e-0107453abaf6

**Published:** 2012-08-09

**Authors:** Christopher A. G. Söderberg, Wietske Lambert, Sven Kjellström, Alena Wiegandt, Ragna Peterson Wulff, Cecilia Månsson, Gudrun Rutsdottir, Cecilia Emanuelsson

The images for Figures 3 and 4 were incorrectly switched. The image that appears as Figure 3 should be Figure 4, and the image that appears as Figure 4 should be Figure 3. The figure legends appear in the correct order. 

